# Resection of the entire first rib for a malignant tumor via the transclavicular approach: a case report and literature review

**DOI:** 10.3389/fsurg.2026.1729706

**Published:** 2026-03-18

**Authors:** Zhiqing Zhao, Xinli Sun, Bing Wang, Taiqiang Yan

**Affiliations:** Department of Orthopedics, Peking University First Hospital, Beijing, China

**Keywords:** first rib tumor, resection, surgical approach, surgical technique, transclavicular approach

## Abstract

**Background:**

Malignant tumors of the first rib are extremely rare. Resection of the entire first rib is surgically challenging and requires an ingenious approach due to the complex anatomical characteristics. Currently, there is no standardized surgical approach. We herein report a case requiring resection of the entire first rib for a malignant tumor via the transclavicular approach and review relevant literature.

**Case presentation:**

A 43-year-old Chinese male suffered from right-sided neck and back pain for 1 month. Imaging studies (chest computed tomography, magnetic resonance imaging, and positron emission tomography-computed tomography) revealed an osteolytic lesion with pathological fracture in the right first rib, suggestive of malignancy. Due to the lesion's proximity to critical neurovascular structures, percutaneous biopsy was not feasible. The patient underwent *en bloc* resection of the entire first rib via the transclavicular approach, which included a V-shaped clavicular osteotomy for exposure, meticulous mobilization and protection of the subclavian vessels and brachial plexus, and subsequent anatomical clavicular reconstruction with a locking plate. The postoperative course was uneventful, with resolution of symptoms and no neurological deficits. Histopathology confirmed lymphoma.

**Conclusions:**

The transclavicular approach provides excellent exposure for the safe and complete resection of first rib tumors, facilitating neurovascular protection and direct visualization. This technique offers a valuable surgical option for selected tumors in this challenging location.

## Background

Tumors of the ribs are uncommon, with those arising from the first rib being exceptionally rare. Resection of first rib tumors poses significant challenges due to limited exposure and the proximity of vital neurovascular structures, including the subclavian vessels and brachial plexus (1). The clavicle overlies the first rib, further complicating surgical access. Owing to the rarity of these tumors, there is no consensus on the optimal surgical approach (2–5). Various techniques have been described, including the transaxillary, transmanubrial, supraclavicular, and combined approaches, each with its own advantages and limitations (6–10). This report presents a case of successful entire first rib resection for a malignant tumor using the transclavicular approach (anterior approach with clavicular transection and reconstruction) and provides a comprehensive review of the literature.

## Case presentation

**Figure 1:**
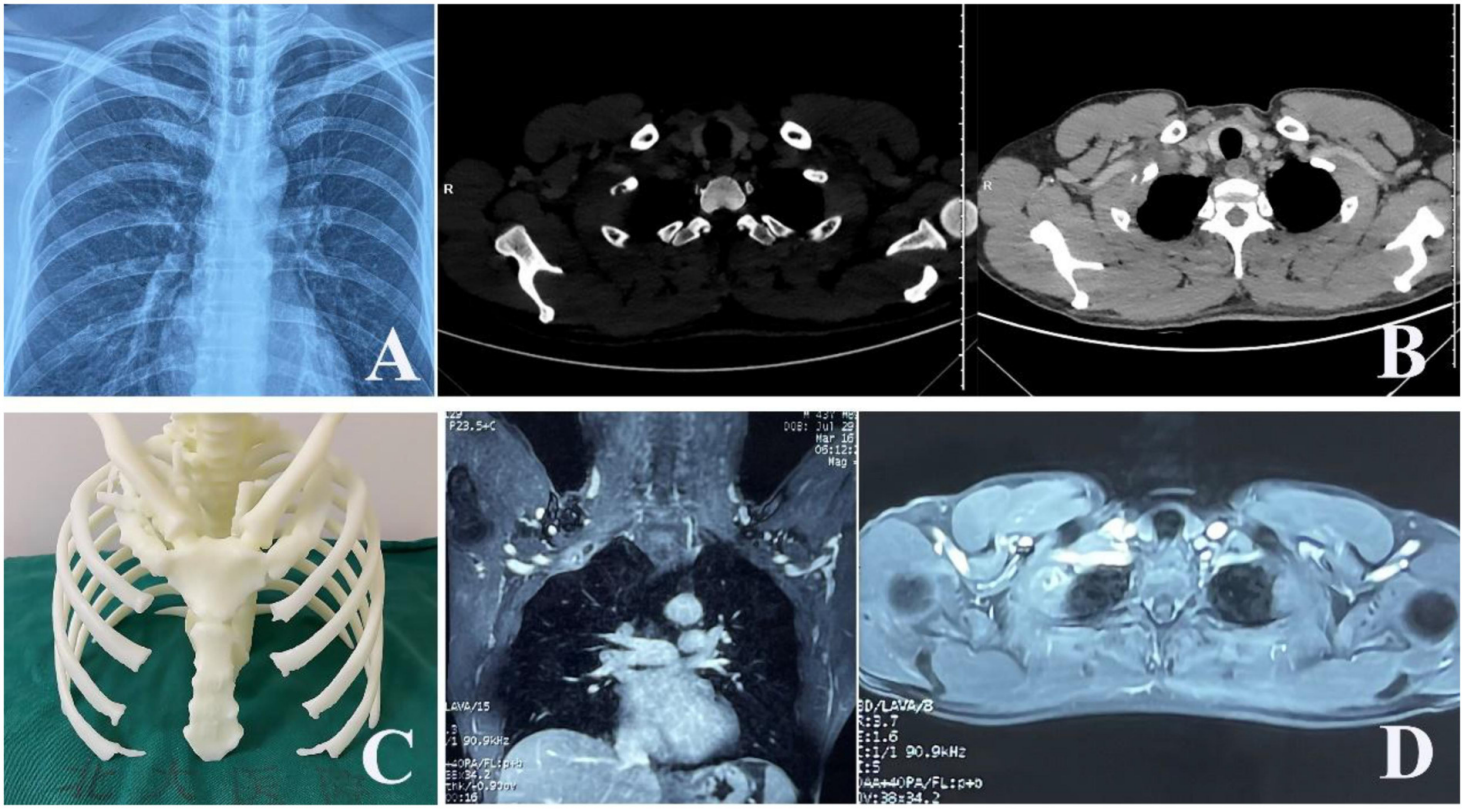
**(A)** An X-ray shows a lytic lesion and fracture in the right first rib; **(B)** a CT scan shows osteolytic destruction in the mid-segment of the right first rib, with soft tissue swelling; **(C)** the 3D printed model based on the CT scan; **(D)** T2-weighted MRI images show high-intensity signals in the middle part of right first rib.

**Figure 2:**
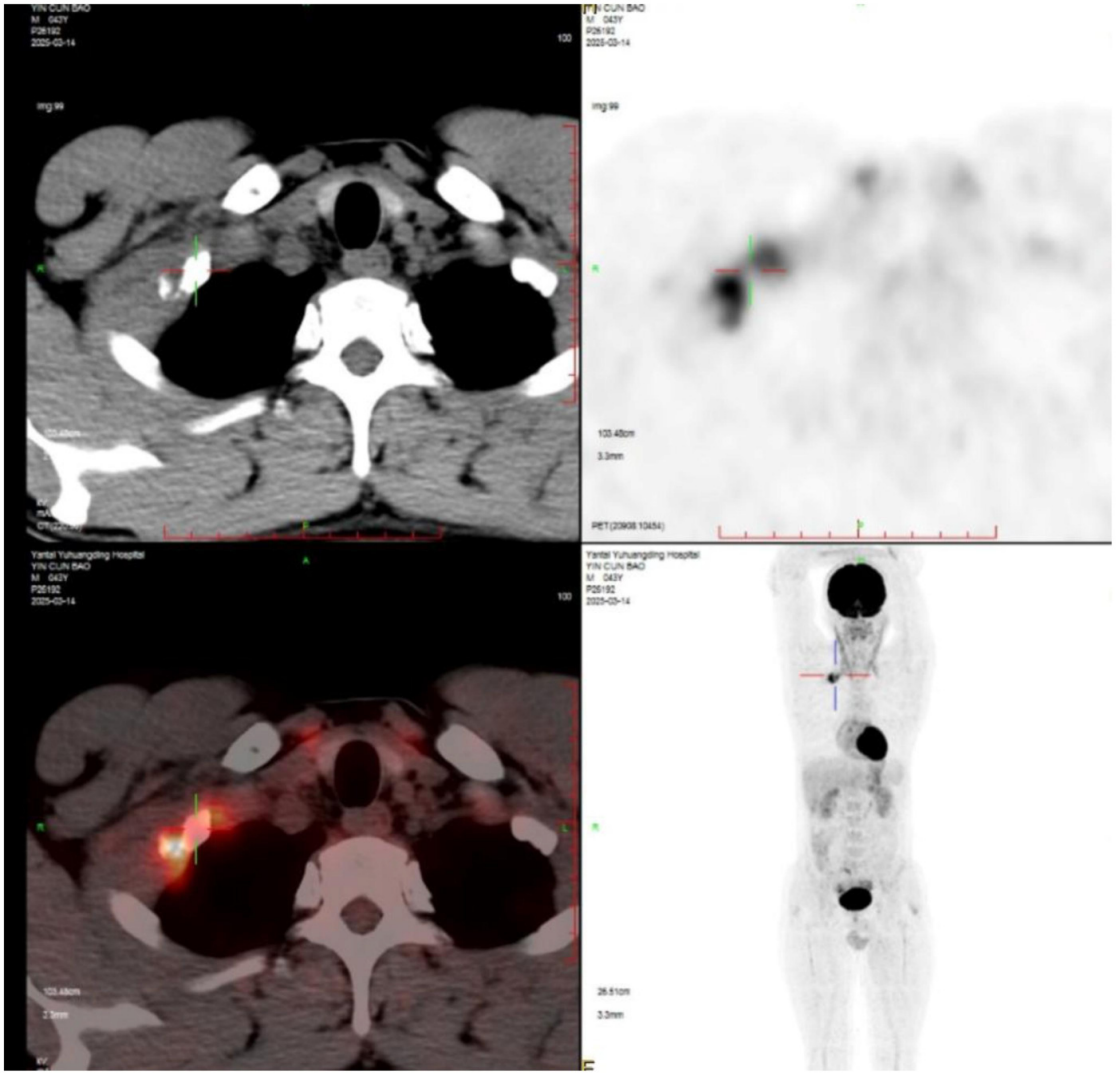
PET-CT demonstrates a single lesion in the middle part of the first right rib with an SUVmax of 11.1.

A 43-year-old, previously healthy man experienced right-sided neck and back pain for 1 month. The physical examination was unremarkable. Laboratory investigations showed no abnormalities. A plain chest radiograph revealed a lytic lesion with an associated fracture in the right first rib (Figure 1A). Subsequent contrast-enhanced chest computed tomography (CT) demonstrated an osteolytic, expansile lesion in the mid-portion of the right first rib with cortical destruction and adjacent soft tissue swelling (Figure 1B). A three-dimensional (3D) printed model based on the CT scan illustrated the first rib lesion and its relationship with the subclavian vessels (Figure 1C). Magnetic resonance imaging (MRI) showed heterogeneous, high signal intensity on T2-weighted sequences within the lesion (Figure 1D). Fluorodeoxyglucose positron emission tomography-computed tomography (PET-CT) identified a solitary hypermetabolic focus in the same location [standardized uptake value maximum (SUVmax) of 11.1] with no other lesions (Figure 2). Given the imaging characteristics suggestive of malignancy and the lesion's inaccessible location for percutaneous biopsy, surgical excision was planned.

### Surgical technique

**Figure 3:**
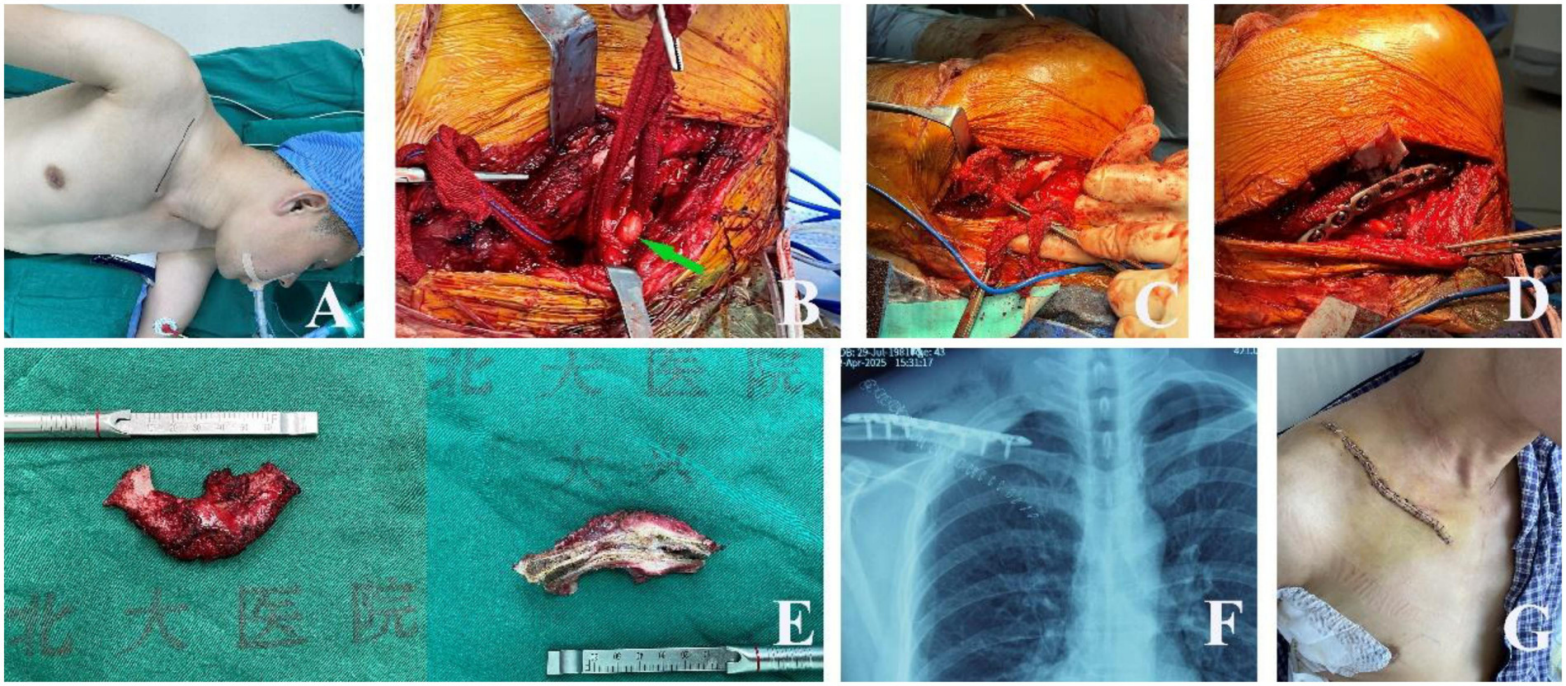
**(A)** The patient is positioned in the left decubitus position, and a right supraclavicular surgical incision is created. **(B)** The brachial plexus is carefully dissected and protected (green arrow). **(C)** The resected first rib is extracted through the infraclavicular space. **(D)** Anatomical reduction of the clavicle is achieved using an anatomically contoured locking plate. **(E)** Gross specimen of the resected first rib and the cross-section shows the tumor within the rib and clear surgical margins. **(F)** A postoperative X-ray confirms stable clavicular fixation. **(G)** A photo of the surgical wound after surgery.

The patient was placed in a left lateral decubitus position under general anesthesia with double-lumen endotracheal intubation. The right cervicothoracic region was prepared and draped. A 12-cm supraclavicular incision was made extending from the sternoclavicular joint to the acromioclavicular joint (Figure 3A). Subcutaneous tissues and the platysma were divided. The clavicular insertion of the sternocleidomastoid muscle was detached. The clavicle was exposed, and a V-shaped osteotomy was performed at its midshaft using a piezosurgical device. This allowed elevation of the medial clavicular segment along with the underlying musculature.

The phrenic nerve was identified coursing on the anterior surface of the anterior scalene muscle and was gently mobilized and protected throughout the procedure. The anterior scalene muscle was then divided close to its insertion into the first rib, taking care to avoid injury to the underlying subclavian artery. The subclavian vein and artery were meticulously dissected free from the rib and encircled with vessel loops. A moist gauze was passed beneath the vascular bundle to facilitate its gentle retraction (Figure 3B).

The brachial plexus trunks were identified posterior to the anterior scalene muscle. The plexus was carefully dissected from the tumor and surrounding tissues. Particular attention was paid to avoiding excessive traction. The middle scalene muscle was detached from the first rib, further exposing the rib posteriorly. A moist gauze was passed under the brachial plexus and used to gently lift the brachial plexus to create a space between the first rib and the brachial plexus (Figure 3B).

With the neurovascular structures protected and retracted, the first rib was fully visualized. The intercostal muscles were then divided. The rib was disarticulated posteriorly at the costotransverse joint and anteriorly at the sternocostal junction using rib cutters. The entire first rib, along with the tumor, was then removed *en bloc* through the infraclavicular space (Figure 3C). A small pleural tear was noted and repaired. A 24-Fr chest tube was inserted through a separate incision in the seventh intercostal space at the midaxillary line for postoperative drainage. The clavicular fragments were anatomically reduced and fixed with a pre-contoured titanium locking plate (Figure 3D). The wound was closed in layers. Finally, the specimen showed a satisfactory macroscopic margin (Figure 3E).

## Postoperative course and follow-up

The patient's postoperative recovery was smooth. The chest tube was removed on postoperative day 3. His preoperative neck and back pain resolved completely. Upon a neurological examination, the right upper limb remained intact, with no evidence of brachial plexus injury, Horner's syndrome, or phrenic nerve palsy. A postoperative X-ray confirmed stable clavicular fixation (Figure 3F). The clavicular wound healed well without infection (Figure 3G). The patient was discharged on postoperative day 10. At the 3-month follow-up, he reported no pain, had full shoulder girdle function, and was satisfied with the cosmetic outcome. A histopathological examination diagnosed diffuse large B-cell lymphoma. The patient was referred to the hematology department for adjuvant therapy.

## Discussion

First rib tumors present a formidable surgical challenge due to the overlying clavicle and the intimate relationship with the subclavian vessels, brachial plexus, and phrenic nerve (1, 11). This anatomical complexity also makes preoperative needle biopsy hazardous and often non-diagnostic (12). Therefore, careful preoperative planning with advanced imaging (CT, MRI, PET-CT, and 3D reconstruction) is of paramount importance to define the tumor's extent and its relationship with adjacent structures (13).

Our case illustrates the utility of the transclavicular approach for achieving the wide exposure necessary for safe, *en bloc* resection of a malignant first rib tumor. The key surgical principles in this region, shared with procedures for Pancoast tumors, cervicothoracic junction tumors, and thoracic outlet syndrome, include the following: (1) systematic identification and protection of the phrenic nerve on the anterior scalene muscle; (2) meticulous dissection and control of the subclavian vessels prior to rib manipulation; and (3) gentle handling and minimal retraction of the brachial plexus cords to prevent neuropraxia (5, 14, 15).

The literature describes several approaches to the first rib, including the transaxillary, transmanubrial, supraclavicular, and combined methods (2–5, 8–10). The transclavicular approach, involving clavicular osteotomy, provides direct anterior access to the thoracic inlet. While not “minimally invasive” in the conventional sense, it offers a wide and safe surgical field that is crucial for vascular control and complete tumor excision, especially for malignancies where *en bloc* resection is the goal. The main drawback is the need for clavicular osteotomy and reconstruction. However, as demonstrated, anatomical fixation with a modern locking plate provides stable union and preserves shoulder girdle function without cosmetic deformity, in contrast to older techniques involving clavicular resection. This approach appears particularly suitable for the following: (1) malignant or locally aggressive benign tumors requiring wide margins; (2) tumors located in the mid-portion of the rib where vascular control is critical; and (3) cases where other approaches (e.g., transaxillary) may offer insufficient exposure for safe dissection.

A literature review of first rib resection was performed after searching the PubMed database. Though not based on a systematic meta-analysis, it summarized reported cases and highlighted that complete first rib resection is rarely performed. Only 12 cases have been reported between 2000 and 2025. Table 1 presents a summary of these previous studies.

**Table 1:** Reported cases of first rib resection in the English literature (2000–2025).

Author(s), year	Sex	Age	Tumor	Approach	Entire rib resection	Complication
Yeow and Hsieh, 2001 (14)	50	F	Hemangioma	Transaxillary, infraclavicular, and supraclavicular approaches	No	Subclavian vessels compressed by hematoma formation
Cheng et al., 2007 (15)	21	F	Aneurysmal bone cyst	Transmanubrial approach	No	None
	42	M	Aneurysmal bone cyst	Transmanubrial approach		None
Masahiro Kitada et al, 2009 (16)	56	M	Chondrosarcoma	Anterior approach (severing the clavicle), thoracoscope, and thoracotomy	No	None
O’Brien et al., 2010 (17)	12	F	Osteochondroma	Supraclavicular and infraclavicular approach	No	Acute thrombosis of the subclavian vein
Kemp et al., 2012 (18)	41	F	Fibrous dysplasia	Transaxillary approach and posterolateral thoracotomy	No	None
Furukawa et al., 2012 (7)	27	F	Fibrous dysplasia	Combined posterior-transmanubrial approach	Yes	None
Medina and Paul, 2016 (5)	M	17	Aneurysmal bone Cyst	Anterior approach (Transclavicular: clavicle was resected and reimplanted)	No	None
Matsunobu et al., 2021 (8)	27	F	Giant cell tumor in the bone	Transmanubrial approach	No	None
Tvedten et al., 2021 (19)	5	F	Osteochondroma	NA	NA	NA
Peng et al., 2023 (9)	26	F	Osteoblastoma	Robotic-assisted wire saw resection	NA	None
	51	M	Epithelioid malignant tumor			None
	21	M	Fibro-osseous lesion			None
Chen et al., 2024 (20)	18	M	Osteochondroma	Trans manubrial approach (L-shaped skin incision)	Yes	Brachial plexus injury
Buero et al., 2025 (10)	18	M	Hemangiomas	Combined approach (video-assisted thoracoscopy and posterior access)	Almost	Asystole

The previously reported transclavicular approach (2, 6, 11) requires the removal of the clavicle, which may offer a good surgical view, but postoperative deformity and functional impairment of the shoulder girdle were also reported. Compared to a previously reported method involving complete clavicular resection for surgical exposure and subsequent reconstruction (5), our approach is relatively minimally invasive.

It is worth mentioning that three-dimensional reconstruction of computed tomography can determine the size, location, and internal structure of the tumor, as well as its relationship with neighboring organs, playing a guiding role in surgical resection planning. These examinations enabled us to delineate the extent of the tissues surrounded by the tumor, thereby informing our decision on the most appropriate surgical approach.

In general, chest wall reconstruction is recommended when at least three ribs are resected or the size of the chest wall defect is ≥10 cm (12, 13). Therefore, chest wall reconstruction was not performed in this patient. A brachial plexus injury did not occur after surgery. The patient recovered gradually after 2 weeks with no residual symptoms. It is important to note that gentle manipulation during exposure of the subclavian tissue, avoidance of excessive stretching, and limited use of electrocoagulation are recommended.

The limitations of this report include its nature as a single case and the inherent selection bias. Larger, multicenter studies are needed to better define the indications and outcomes of different surgical approaches for first rib tumors.

In conclusion, the transclavicular approach with clavicular osteotomy and reconstruction is a valuable and safe technique for resecting challenging tumors of the first rib. It provides excellent exposure, protects critical neurovascular structures, and leads to complete oncological resection.

## Data Availability

The original contributions presented in the study are included in the article/Supplementary Material, further inquiries can be directed to the corresponding author.
